# *In vivo* Bioimaging as a Novel Strategy to Detect Doxorubicin-Induced Damage to Gonadal Blood Vessels

**DOI:** 10.1371/journal.pone.0023492

**Published:** 2011-09-09

**Authors:** Hadas Bar-Joseph, Irit Ben-Aharon, Moran Tzabari, Galia Tsarfaty, Salomon M. Stemmer, Ruth Shalgi

**Affiliations:** 1 Department of Cell and Developmental Biology, Sackler Faculty of Medicine, Tel-Aviv University, Israel; 2 Davidoff Center, Rabin Medical Center, Institute of Oncology, Petah-Tiqva, Israel; 3 Department of Diagnostic Imaging, Sheba Medical Center, Tel Hashomer, Israel; Institute for Medical Biomathematics, Israel

## Abstract

**Introduction:**

Chemotherapy may induce deleterious effects in normal tissues, leading to organ damage. Direct vascular injury is the least characterized side effect. Our aim was to establish a real-time, *in vivo* molecular imaging platform for evaluating the potential vascular toxicity of doxorubicin in mice.

**Methods:**

Mice gonads served as reference organs. Mouse ovarian or testicular blood volume and femoral arterial blood flow were measured in real-time during and after doxorubicin (8 mg/kg intravenously) or paclitaxel (1.2 mg/kg) administration. Ovarian blood volume was imaged by ultrasound biomicroscopy (Vevo2100) with microbubbles as a contrast agent whereas testicular blood volume and blood flow as well as femoral arterial blood flow was imaged by pulse wave Doppler ultrasound. Visualization of ovarian and femoral microvasculature was obtained by fluorescence optical imaging system, equipped with a confocal fiber microscope (Cell-viZio).

**Results:**

Using microbubbles as a contrast agent revealed a 33% (P<0.01) decrease in ovarian blood volume already 3 minutes after doxorubicin injection. Doppler ultrasound depicted the same phenomenon in testicular blood volume and blood flow. The femoral arterial blood flow was impaired in the same fashion. Cell-viZio imaging depicted a pattern of vessels' injury at around the same time after doxorubicin injection: the wall of the blood vessels became irregular and the fluorescence signal displayed in the small vessels was gradually diminished. Paclitaxel had no vascular effect.

**Conclusion:**

We have established a platform of innovative high-resolution molecular imaging, suitable for *in vivo* imaging of vessels' characteristics, arterial blood flow and organs blood volume that enable prolonged real-time detection of chemotherapy-induced effects in the same individuals. The acute reduction in gonadal and femoral blood flow and the impairment of the blood vessels wall may represent an acute universal doxorubicin-related vascular toxicity, an initial event in organ injury.

## Introduction

Chemotherapy may exert deleterious effects on normal tissues, leading to organ damage. It is now apparent that chemotherapy administration contributes to adverse vascular conditions that lie at the basis of a heterogeneous group of disorders. The precise pathogenesis of these toxic effects has not been elucidated. Chemotherapy-induced vascular toxicity may cause acute cardiovascular complications that could be the initiation seed for progression of long-term diseases such as atherosclerosis and for increased risk of vascular events. The potential vascular insult characteristics of several chemotherapeutic agents have been studied, and doxorubicin was shown to stimulate apoptosis in endothelial cells [1]–[3].

Doxorubicin, an anthracycline that is a cornerstone of many chemotherapeutic protocols, is used for treating a wide spectrum of malignancies. Cardiomyocytes and endothelial cell-lines exposed to doxorubicin *in vitro,* exhibited a dose-related apoptosis that may occur due to generation of free radicals [4]–[9]. Experiments in intact aortic rings demonstrated that acute exposure (<30 minutes) to doxorubicin results in generation of oxygen radicals in endothelial cells via a flavoprotein containing oxido-reductase [10]. Organ culture studies as well as mammalian models have also shown that doxorubicin exerts harmful effects on vascular endothelium, leading to impaired vasodilatory response of arteries [1]–[3], [10], [11]. It appears that administration of a single dose of doxorubicin in rabbits is associated with rapid deterioration of endothelium-dependent and independent vascular responses. Furthermore, administration of doxorubicin in humans is associated with acute reduction of flow-mediated dilatation in the brachial arteries and of nitric oxide level in the plasma [11].

High-frequency ultrasound with enhanced contrast agents enables *in vivo* imaging and analysis of blood perfusion. Microbubbles are a contrast agent that enhances the acoustic signal of blood in the circulation; they are small enough to move freely through the bloodstream, and are used as markers for visualization and quantification of regional microvasculature [12], [13]. Fibred confocal fluorescence microscopy (FCFM) was designed for *in vivo* imaging of fluorescent signals in living animals. The FCFM with its optical mini-probes enables *in vivo* fluorescent visualization of microvasculature with a minimal invasive intervention [14].

We have set up a platform of live, high-resolution molecular mice imaging, suitable for capturing vessels' characteristics, arterial blood flow and organs blood volume. This imaging setup enables us to detect acute, real-time, treatment-induced effects within the same individuals and follow them over a period of time. Using both imaging tools, we could observe that doxorubicin had an effect on blood vessels already 3 minutes after administration. The acute reduction (of 33% from baseline values) in gonadal (ovarian or testicular) and femoral blood flow and the impairment of the blood vessels wall may represent an acute universal doxorubicin-related vascular toxicity, an initial event in organ injury.

## Materials and Methods

Animal care and all experiments were in accordance with institutional guidelines and were approved by the Institutional Animal Care and Use Committee, Sackler Faculty of Medicine, Tel-Aviv University, ID number M-09049.

### Animals

ICR mature male and female mice (7–8 weeks old; Harlan Laboratories, Jerusalem, Israel) were housed in air conditioned, light controlled animal facilities of the Sackler faculty of Medicine in Tel-Aviv University.

### Chemotherapy

Doxorubicin (8 mg/kg, Adriamycin; Teva, Israel), paclitaxel (1.2 mg/kg, Medexel; Taro, Israel) or saline were injected intravenously (IV) into the tail vein at a volume of 100 µl.

### Ultrasound imaging

The molecular bioimaging platform enables real-time evaluation of the same individual over time, and hence each mouse serves as its own control.

#### Preparing the animals for imaging

Mice were anesthetized with isoflurane (Nicholas Piramal India Limited, India; 5% in oxygen for induction, 1–3% for maintenance at a rate of 1liter/minute). Hemodynamic measurements were performed continuously throughout the experiments. Mice were positioned on a MousePad (part of the VisualSonics Vevo Integrated Rail System II) equipped with integrated heater and ECG electrodes (IndusInstruments,Houston,TX). 4 legs were secured to ECG pads, with mediation of electrode cream (Signa cream; Parker Laboratories Inc., Fairfield, NJ, USA), to allow continuous monitoring of respiration rate. Respiration rate was maintained constant (varied between 20–40 breaths/minute in the cohort), body temperature was maintained at 37.5°C.

A 30-gauge, 1/2-inch needle attached to 1 ml syringe was inserted into the tail vein for IV administration of both contrast agent and either doxorubicin, paclitaxel or saline. Dorsal and groin fur was removed by a depilatory cream (Veet, Reckitt Benckiser, Bristol, UK). Pre-warmed ultrasound gel (Aquasonic, Parker Laboratories Inc, Fairfield, NJ, USA) was used as a coupling agent between the ultrasound scan-head and the skin.

Mice gonads or femoral arteries were viewed by the color mode of the high-resolution ultrasound (Vevo 2100; Visual Sonics, Toronto, Canada), with the transducer (MicroScan MS 550D; 22–55 MHz) held immobilized, in-position by the VisualSonics Vevo Integrated Rail System II.

To reduce variability, image parameters remained constant throughout the experiment (i.e., focus and depth optimized for each animal at the beginning of the experiment and the point of monitoring was fixed through the entire experiment). The same scan plane approximation, determined by anatomic markers, was used in all experiments.

#### Ultrasound contrast imaging of the ovarian blood volume

Gas-filled (nitrogen and perflurobutane) microbubbles (Definity, Lantheus Medical Imaging, MA, USA; 1.1–3.3 µm diameter; maximum concentration of 1.2×10^10^ microbubbles/ml), an ultrasound contrast agent, were injected IV into mice. Prior to each imaging, the microbubbles were reconstituted by a 15 second gentle stir, diluted with saline (1∶1) and injected IV into the tail vein in 80 µl bolus. Ovaries were viewed at the contrast mode and their blood volume, which was reflected by the microbubbles intensity, was determined by the appropriate VisualSonics software. The baseline rate of ovarian blood volume was quantified after a short stabilization period (at which the mouse was positioned) by analyzing the intensity of the contrast agent during a 50 second image recording period (referred to as “cine loop”), as previously described [12], [13]. Mice were then injected IV with either doxorubicin (n = 7), paclitaxel (n = 8) or saline (n = 7; control). Rate of ovarian blood volume was re-quantified 3, 10 and 20 minutes after the IV injection of either chemotherapy or saline. The circulating microbubbles were destroyed between injections by the pulse-wave (PW) Doppler mode (100% power).

#### PW Doppler measurement of blood flow in testicular or femoral vessels

Testicular blood volume and flow as well as femoral arterial blood flow was viewed at the PW Doppler mode using the appropriate VisualSonics software. Following a short stabilization period, a baseline femoral or testicular arterial blood flow was recorded by the PW Doppler mode during a 50 second cine loop and quantified by analyzing the Velocity-Time Integral (VTI) [15]-[18]. When the PW Doppler mode curve is integrated, it yields a VTI that indicates the distance the blood travels during a certain cardiac circle. Mice were then injected IV with either doxorubicin (n = 12, n = number of imaged arteries), paclitaxel (n = 14) or saline (n = 10) for recording femoral arteries blood flow, or doxorubicin (n = 8) or saline (n = 7) for recording testicular artery blood flow. The arterial blood flows were monitored continuously for 20 minutes, and recorded and analyzed at various time points post injection. To note, this methodology was not applicable for measuring ovarian blood flow technically due to the deep location and size of the ovaries.

#### Image analysis

Acquired contrast (ovarian blood volume) and PW Doppler (testicular and femoral arterial blood flow) cine loops were digitally stored and pooled for off-line analysis. Analysis of all four ovary blood volume cine loops captured for each mouse, was performed at a fixed region of interest (ROI), and the data was presented as an Excell curve, provided by the VisualSonics software. A trend line (mean of 20 points) was added to each curve and the Δ between the microbubbles “first pass” value (highest point) and the reference value (lowest point) was calculated. Analysis of the femoral arterial blood flow and testicular blood flow for each PW Doppler cine loop was performed by adding an automatic frequency trace to each cine loop and acquiring 2–4 regions of VTI, in accordance to the breathing signal. Values of post-treatment imaging (calculated proportionally for microbubbles cine loops; mean value for PW Doppler VTIs) were normalized according to pre-treatment imaging values of each mouse (defined as 100%). Saline-injected mice were standardized to 100% as a reference to chemotherapy-injected mice. Non normalized values are presented in the supplement.

### Fibred confocal fluorescence microscopy (FCFM) imaging

FCFM (Cell-viZio; Mauna Kea Technologies, Paris, France) was developed for *in situ* and *in vivo* imaging [14], [19]. Its technology is based on the excitation of either intrinsic or extrinsic fluorescent molecules in the examined tissue. The Cell-viZio is composed of a laser scanning unit LSU-488 (FibroScan) that uses a laser source with a wavelength of 488 nm and of a ProFlex microprobe (mini0/30). FCFM imaging of ovarian or femoral vessels during and following doxorubicin, paclitaxel or saline administration was performed in mice under general anesthesia (100 mg/kg ketaset, Fort Dodge Animal Health, IA, USA and 6 mg/kg XYL-M2, Biove Laboratories, France). For *in vivo* ovary imaging the abdominal skin above the ovary was incised. For femoral arterial vessels imaging, the skin was incised below the groin. FITC-dextran at a volume of 100 µl (10 mg/ml; FD2000S, MW 2000000 Dalton; Sigma) was administered IV to facilitate the FCFM visualization of the microsvascular network. A 488-nm wavelength laser was used for all dynamic observations. The emitted fluorescence was filtered (500 to 650 nm) prior to detection by a detector housed in the main unit. The images were then reconstructed and viewed on a real-time display at 12frames/second. A baseline recording of the flow was obtained during a short stabilization period, following mouse positioning and before an IV injection of doxorubicin (n = 19), paclitaxel (n = 7) or saline (n = 9). FITC-dextran flow was monitored continuously for 20 minutes after the injections. Blood vessels were categorized according to their diameter, and were defined *a priori* as small (<15 µm), and large (>15 µm).

### Statistical analysis

SPSS 10.0 software (SPSS Inc, Chicago, IL, USA) was used for statistical analysis. T-TEST with two-tailed distribution and two-sample unequal variance was employed for assessing the degree of change in the ovarian blood volume experiment. Two-way analysis of variance (ANOVA) with repeated measures was employed for assessing the degree of change in blood flow in the femoral arterial blood flow or testicular blood flow experiment. Results were considered statistically significant at P<0.01. Results are given as mean±SD.

## Results

### *In vivo* ovarian ultrasound imaging

**Figure 1:**
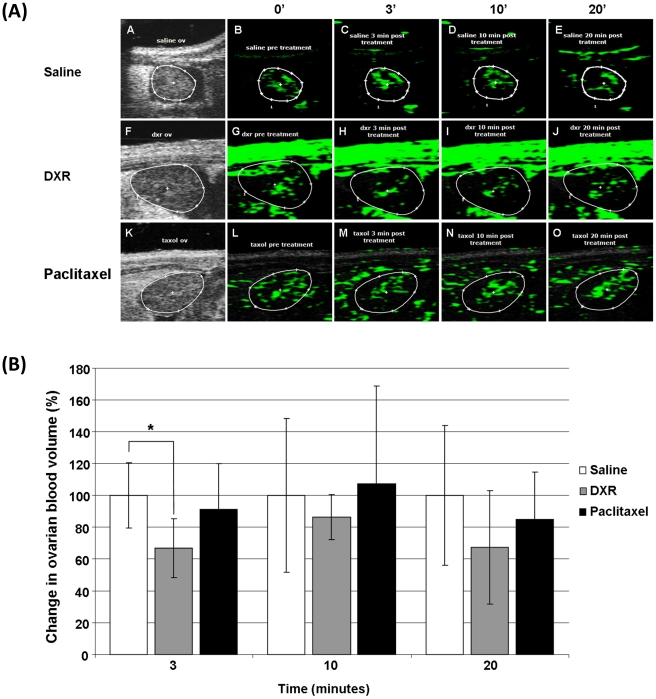
Ultrasound contrast imaging of the ovarian blood volume. A. Representatives images of analyzed microbubbles contrast agent intensity performed at a fixed region of interest (ROI). The Vevo ultrasound depicted a detailed imaging of the blood volume of mouse ovary before and following administration of either saline (a–e) doxorubicin (f–j) or paclitaxel (k–o). Anatomy of the ovary in contrast mode (a,f,k); analysis of contrast agent intensity determined using the appropriate VisualSonics software (b–e, g–j, l–o). The green colors indicate the presence of an enhanced contrast agent). Baseline ovary blood volume (pre-treatment; b,g,l). Ovary blood volume 3 minutes (c,h,m), 10 minutes (d,i,n) and 20 minutes (e,j,o) following the IV injection of either chemotherapy or saline. B. Graphic representation of ovarian blood volume 3, 10 and 20 minutes after doxorubicin (n = 7), Paclitaxel (n = 8) or saline (n = 7) injection indicating a decrease in ovarian blood volume of doxorubicin treated mice (* P<0.01). Saline-injected mice were standardized to 100% as a reference to chemotherapy-injected mice. Results are presented as mean±SD. The data obtained from the images as presented in A, was presented as an Excell curve provided by the VisualSonics software.

The Vevo 2100 ultrasound depicted detailed images of mouse ovarian blood volume before and after administration of saline (Fig. 1A b-e, green) or chemotherapeutic agents (Fig. 1A g-j, l-o, green), indicating a 33% decrease in ovarian blood volume already 3 minutes after doxorubicin injection (Fig. 1B; P<0.01) that was partially recovered 10 and 20 minutes after treatment to a degree that was not statistically different from the ovarian blood volume in control mice at the same time points (Fig. 1B). The difference in ovarian blood volume between paclitaxel injected mice and control mice was not statistically significant at any time points (Fig. 1B).

### *In vivo* testicular and femoral ultrasound imaging

**Figure 2:**
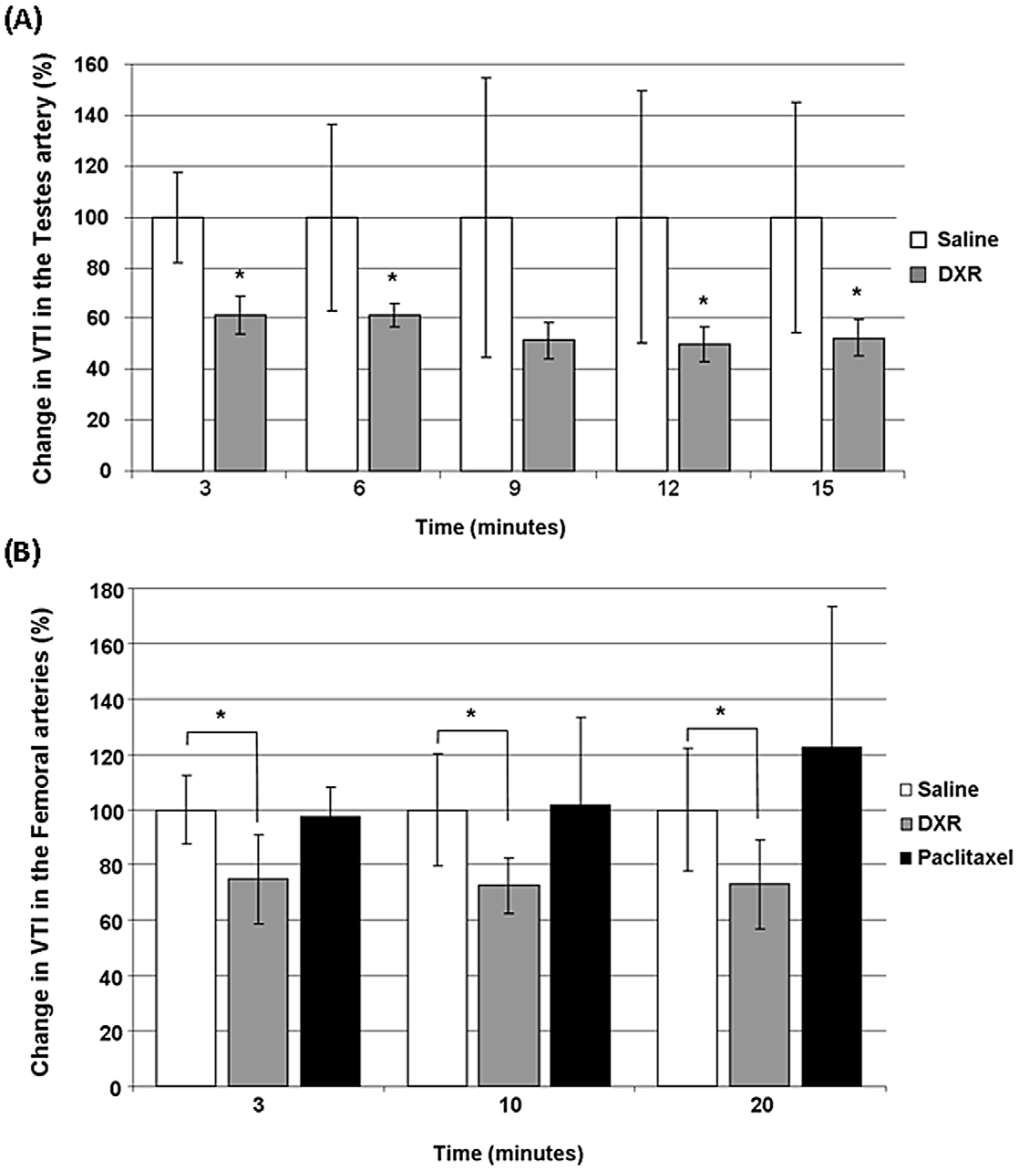
Pulse-wave Doppler measurement of testicular and femoral arterial blood flow. Using PW Doppler mode, blood flow was measured and quantified by analyzing Velocity-Time Integral (VTI) using the appropriate VisualSonics software. PW Doppler blood flow was continuously monitored before and following doxorubicin administration. Saline-injected mice were standardized to 100% as a reference to chemotherapy-injected mice. Results are presented as mean±SD. The data obtained from the images as presented in figure 1A, was presented as an Excell curve provided by the VisualSonics software. A. Graphic representation of testicular blood flow 3, 6, 9, 12 and 15 minutes after doxorubicin (n = 8) or saline (n = 7) injection indicating a rapid and constant fall in testicular blood flow of doxorubicin treated mice (* P<0.01). B. Graphic representation of femoral blood flow volume 3, 10 and 20 minutes after doxorubicin (n = 12), Paclitaxel (n = 14) or saline (n = 10) injection indicating a rapid and constant fall in femoral blood flow of doxorubicin treated mice (* P<0.01).

The effect of doxorubicin or paclitaxel on the testicular blood flow and the femoral arterial blood flow was assessed by measuring VTI with the PW Doppler. Both the testicular and the femoral vessels depicted the same fashion: A constant rapid fall in the testicular blood flow (40% decrease, P<0.01) and in the arterial femoral blood flow (23% decrease, P<0.01) was evident already 3 minutes after doxorubicin administration and lasted even 20 minutes after treatment (Fig. 2A+B). Paclitaxel had no effect on femoral arterial blood flow (Fig. 2B). Non-normalized figures are presented in figures S1B, S2A, S2B.

### Imaging of ovarian and femoral microvasculature

**Figure 3:**
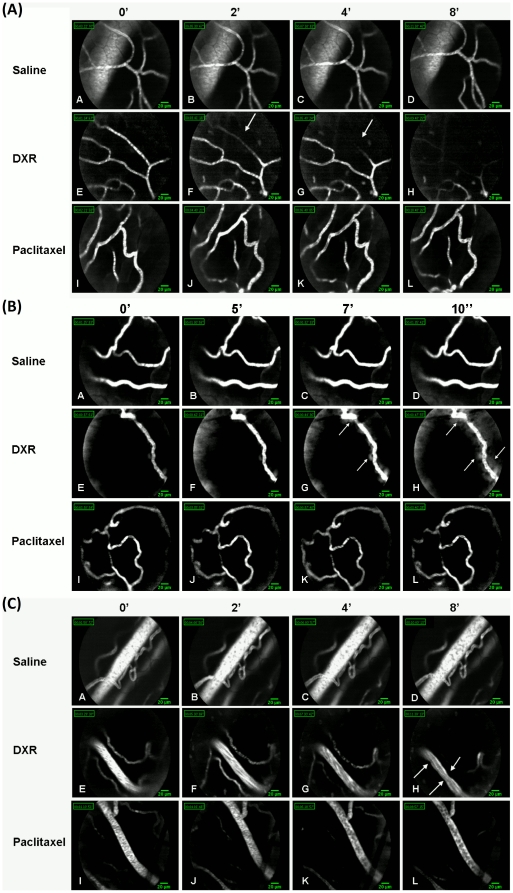
Ovarian and femoral microvasculature imaging. Representative images of FITC-dextran fluorescence signal in small (<15 µm diameter) and large (>15 µm diameter) blood vessels. A continuous recording of ovarian and femoral microvasculature, of mice injected with FITC-dextran (100 µl; 10 mg/ml) was obtained before and during an IV injection of doxorubicin (n = 19), paclitaxel (n = 8) or saline (n = 9). A. The acute vascular effect observed by FCFM in the small vessels, started approximately 2 minutes after doxorubicin administration (f, arrow), characterized by a constant narrowing that caused a complete disappearance of the fluorescent signal from the imaged blood vessels, with no apparent recovery during the next 8 minutes of real time imaging (g,h, arrows). B. In some experiments, a “blurred” area was evident over the blood vessel wall of doxorubicin-injected mice (g,h, arrows) a few seconds after doxorubicin administration, suggesting a potential FITC leakage. C. Large vessels exhibited distortion of the vessel wall, manifested by the appearance of irregular wall surface (h, arrows), 4 minutes after doxorubicin administration.

We have characterized, the network of the ovarian and femoral blood vasculature, according to vessels diameter (small, <15 µm; large, >15 µm), by FCFM in mice injected with FITC-dextran. Small vessels became narrower approximately 2–5 minutes after administration of doxorubicin and continued so until a complete disappearance of the FITC-dextran fluorescent signal at around 8 minutes after treatment (Fig. 3A e-h, arrows; Video S1). In several mice, haziness of the perivascular region could be spotted, a few seconds after doxorubicin administration (Fig. 3B g,h, arrows; Video S2), pointing at a possible leakage from the blood vessel to the surrounding tissues. The wall of large blood vessels became irregular approximately 4 minutes after doxorubicin administration (Fig. 3C, e-h, arrows). Blood vessels of paclitaxel-injected mice (Fig. 3A i–l, 3B i–l, Fig. 3C i–l) resembled those of saline-injected mice (Fig. 3A a–d, 3B a–d, Fig. 3C, a–d), where no changes were observed in the structure or baseline dynamics of blood vessels all throughout the measurement period. No fluorescence signal was evident following administration of doxorubicin in mice that were not injected previously with FITC-dextran.

## Discussion

Of all the chemotherapy-induced side effects, the direct vascular injury is the least characterized. The vascular endothelium is an essential barrier that protects the tissues integrity, regulates the homeostasis of water and solvents between the plasma and the tissues and plays a role in the regulation of arterial vasomotor tone. Impairment of the vascular endothelium may result in disintegration of the blood vessel wall and leakage of fluids from the blood into the extracellular matrix, compromising organ function. Studies addressing the vascular toxicity of certain chemotherapeutic agents indicated that they were more toxic to endothelial cells than to tumor cells [20]. Acute chemotherapy-induced vascular damage, reflected as an increase in plasma level of von Willibrand factor and in the intima-media thickness of the carotid artery, was studied *in vivo* in patients suffering from testicular cancer and treated with cisplatin-based chemotherapy [21].

Doxorubicin is used for treating a wide spectrum of malignancies, and hence serves as a prototype in our study. Cardiotoxicity, the most characterized deleterious effect of doxorubicin toxicity, is cumulative dose-related [22]. It has been formerly implied that endothelial damage may contribute to this pathogenesis; in acute cases, the patient may suffer from hypotension, tachycardia and arrhythmia, while an increased cumulative dose of doxorubicin can cause congestive heart failure [23], [24]. It has already been documented that doxorubicin-treated rats develop marked ascites [5] and that the *in vitro* permeability to albumin of bovine pulmonary artery endothelial cells monolayer, 24 hours after exposure to clinically relevant concentrations of doxorubicin, was 10 fold higher than that of control cells [25]. Several studies have confirmed that doxorubicin induces oxidative stress, a condition known to be toxic to endothelial cells, leading to loss of their barrier trait [26], [27]. Few *ex vivo* studies have explored the effect of doxorubicin on tissues excised from doxorubicin-injected animals. An impaired endothelial-dependent vasodilatory response to acetylcholine or adenosine was observed in rabbit and rat models with doxorubicin–induced cardiomyopathy [5], [28]. Brachial artery reactivity, a marker for endothelial vasodilatation function, detected by high-resolution ultrasound, was decreased in human patients that received at least 300 mg/m^2^ of doxorubicin (or daunorubicin) compared to control patients [11]. Furthermore, brachial artery flow-mediated dilation in patients undergoing doxorubicin based chemotherapy was markedly attenuated after a single dose of doxorubicin [10]. Recently, it has been shown by phase-contrast cardiovascular magnetic resonance measurements of PW velocity and aortic distensibility that anthracyclins induce a significant increase in thoracic aorta stiffness in patients receiving anthracyclins compared to age and sex-matched controls [26].

We have previously studied the effect of doxorubicin on mice ovaries, manifested by reduced ovulation rate and ovarian size, as observed by high resolution MRI [29]. Our results indicated an acute insult to the ovary, reflected by the presence of peri-ovarian edema, encouraged us to further investigate the potential acute vascular effect induced by doxorubicin administration [29].

In the current study, ovarian blood volume was visualized by measuring the baseline concentration of injected microbubbles at the designated blood vessels and the concentration throughout the chemotherapeutic treatment. The use of ultrasound imaging in conjunction with enhanced contrast agents was chosen over the use of PW Doppler as it allows assessment of tissue blood perfusion in a larger ROI than PW Doppler and has a higher resolution capability, both are essential in imaging of the small sized vessels of the ovary. Detailed studies using intravital microscopy have shown that microbubbles with a diameter of less than 5 µm are small enough to allow their free movement through the bloodstream, remain confined to the vasculature and are cleared from the blood in about 15 minutes [30]. This methodology does not disrupt tissue dynamics and physiological processes, and allows studies with imaging at multiple time points. Both methods- ultrasound imaging in conjunction with enhanced contrast agents and PW Doppler were compared and validated [12], [13].

A significant acute reduction in ovarian blood volume was observed already 3 minutes after an IV injection of doxorubicin. To explore whether this phenomenon is unique to the ovary, we further studied the other gonad. We examined the vascular effect in testes of male mice treated with doxorubicin. Here due to the superficial location and dimension of the testicular vasculature we employed the PW Doppler and revealed the same pattern of vascular effect as in the ovaries. Subsequently, to further assess whether this was an exclusive gonadal vascular effect or a generalized effect appears in vessels of non end-organs, the femoral vasculature was imaged as a reference. The significant reduction in femoral arterial blood flow that remained compromised throughout the experiment indicated a generalized phenomenon. It is estimated that the non-significant decrease in ovarian blood volume 10 and 20 minutes after doxorubicin administration is due to technical reasons: since the ovary is located within the peritoneal cavity, its blood flow measurement is affected partially by the bowel movements. The measurements in the later time points demonstrated a trend that was not statistically significant probably due to above reason. The testicular blood flow is not affected by those factors and hence is better displayed. Nevertheless, we can postulate upon the testicular blood flow dynamics that resembled those of the femoral vasculature that the same pattern applies also for the ovarian vasculature, supported by the acute significant decrease in blood volume. The acute vascular effect of the testicular vessels may be attributed also by the unique microcirculation of the testes. This architecture prones the testes to pathophysiological states as varicocele, but also potentially to exogenous toxicants. Former studies have characterized this distinctive vasculature [31], [32].

FCFM, equipped with laser scanning confocal technology, enables real-time tracing of fluorescent agents within deep tissues and produces smooth-motion video sequences of blood vessels *in vivo.* It is used for studying the acute effect of the drug *in situ* [19]. We used FCFM, as previously reported [14] to study the acute effect of doxorubicin on the blood vessels of the regional microvasculature. The acute vascular effect observed by FCFM that started approximately 2 minutes after doxorubicin administration, differed according to blood vessels size: small vessels (<15 µm diameter) suffered a constant narrowing already 2 minutes after doxorubicin injection and demonstrated a gradual diminishment of the fluorescence signal from the imaged blood vessels, with no apparent recovery during the next 8 minutes of real-time imaging. In some experiments, “blurred” areas could be spotted over the blood vessels walls, suggesting potential FITC-dextran leakage. This phenomenon was less prominent in large vessels (>15 µm diameter) that exhibited distortion of the blood vessel wall, manifested by the appearance of irregular wall surface. Paclitaxel, a different type of chemotherapeutic agent, did not cause any vascular impairment in mice that could be evaluated by either ultrasound or FCFM. We can therefore deduce that the observed vascular toxicity is characteristic of doxorubicin.

In conclusion, we describe herein an innovative *in vivo* imaging system capable of real-time capturing of chemotherapy-induced acute vascular impairment in the same individual mouse throughout an extended period of time. The acute reduction in gonadal blood volume and femoral arterial blood flow, and the impairment of the blood vessels' wall may represent an acute universal doxorubicin-related vascular toxicity, an initial event in organ injury. The vascular impairment was sometimes followed by altered barrier function of the blood vessels' wall. The observed phenomenon was significant for doxorubicin and was not evident in paclitaxel treated mice.

The established experimental platform may serve for future evaluation of potential agents designated to prevent doxorubicin-induced vascular toxicity. Evidence for the role of several mediators (as endothelial nitric oxide synthase (eNOS), NADPH etc.) in the pathogenesis of doxorubicin-induced endothelial dysfunction has already been proposed in previous studies [10], [25], [33]. This platform may also be used to study in real-time imaging the vascular effect of tyrosine kinase inhibitors (TKIs) routinely used in the oncologic setting, that may have a potential vascular impact as well as acute potential vascular effect of targeted therapies. Elucidating the mechanism that lies at the core of vascular toxicity might be useful in discovering biological keys needed for decreasing the long term potential vascular complications in cancer survivors.

## Supporting Information

Figure S1 **Ultrasound contrast imaging of the ovarian blood volume.** B. Graphic representation of ovarian blood volume 3, 10 and 20 minutes after doxorubicin (n = 7), Paclitaxel (n = 8) or saline (n = 7) injection indicating a decrease in ovarian blood volume of doxorubicin treated mice (* P<0.01). Results are presented as mean±SD of non-normalized values (saline injected mice were not standardized as 100%). The data obtained from the images as presented in A, was presented as an Excell curve provided by the VisualSonics software.
(TIF)

Figure S2 **Pulse-wave Doppler measurement of testicular and femoral arterial blood flow.** Using PW Doppler mode, blood flow was measured and quantified by analyzing Velocity-Time Integral (VTI) using the appropriate VisualSonics software. PW Doppler blood flow was continuously monitored before and following doxorubicin administration. Results are presented as mean±SD of non-normalized values (saline injected mice were not standardized as 100%). The data obtained from the images as presented in figure 1A, was presented as an Excell curve provided by the VisualSonics software. A. Graphic representation of testicular blood flow 3, 6, 9, 12 and 15 minutes after doxorubicin (n = 8) or saline (n = 7) injection indicating a rapid and constant fall in testicular blood flow of doxorubicin treated mice (* P<0.01). B. Graphic representation of femoral blood flow volume 3, 10 and 20 minutes after doxorubicin (n = 12), Paclitaxel (n = 14) or saline (n = 10) injection indicating a rapid and constant fall in femoral blood flow of doxorubicin treated mice (* P<0.01).
(TIF)

Video S1 **Ovarian and femoral microvasculature imaging.** Representative movies of FITC-dextran fluorescence signal in small (<15 µm diameter) blood vessels. A continuous recording of ovarian and femoral microvasculature, of mice injected with FITC-dextran (100 µl; 10 mg/ml) was obtained starting at the time of IV injection of doxorubicin. Small blood vessels exhibited a constant narrowing approximately 2–5 minutes after doxorubicin injection, up to a complete disappearance of the FITC-dextran fluorescent signal, 8 minutes after treatment.
(AVI)

Video S2 **Ovarian and femoral microvasculature imaging.** Representative movies of FITC-dextran fluorescence signal in small (<15 µm diameter) blood vessels. A continuous recording of ovarian and femoral microvasculature, of mice injected with FITC-dextran (100 µl; 10 mg/ml) was obtained starting at the time of IV injection of doxorubicin. Some small blood vessels exhibited a "blurred" area over the vessel wall, suggesting a potential FITC-dextran leakage.
(MPEG)
