# Supplementary figures and images for: In vivo Bioimaging as a Novel Strategy to Detect Doxorubicin-Induced Damage to Gonadal Blood Vessels
Source: PLoS One. 2011 Sep 9;6(9):e23492. doi: 10.1371/journal.pone.0023492 (PMC3170286; doi:10.1371/journal.pone.0023492)

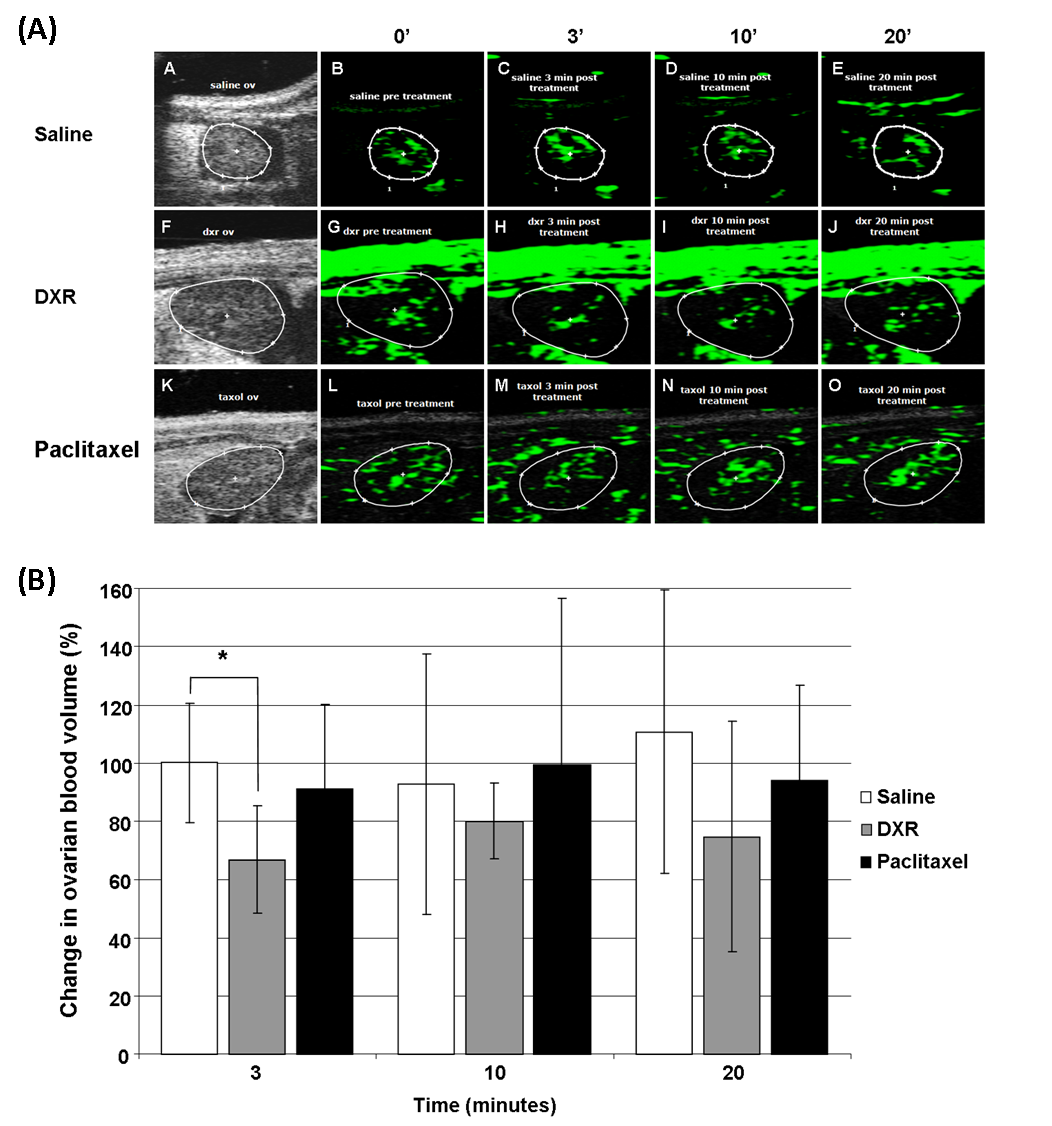

Supplement: Figure S1 — Ultrasound contrast imaging of the ovarian blood volume. B. Graphic representation of ovarian blood volume 3, 10 and 20 minutes after doxorubicin (n = 7), Paclitaxel (n = 8) or saline (n = 7) injection indicating a decrease in ovarian blood volume of doxorubicin treated mice (* P<0.01). Results are presented as mean±SD of non-normalized values (saline injected mice were not standardized as 100%). The data obtained from the images as presented in A, was presented as an Excell curve provided by the VisualSonics software. (TIF) [file pone.0023492.s001.tif]

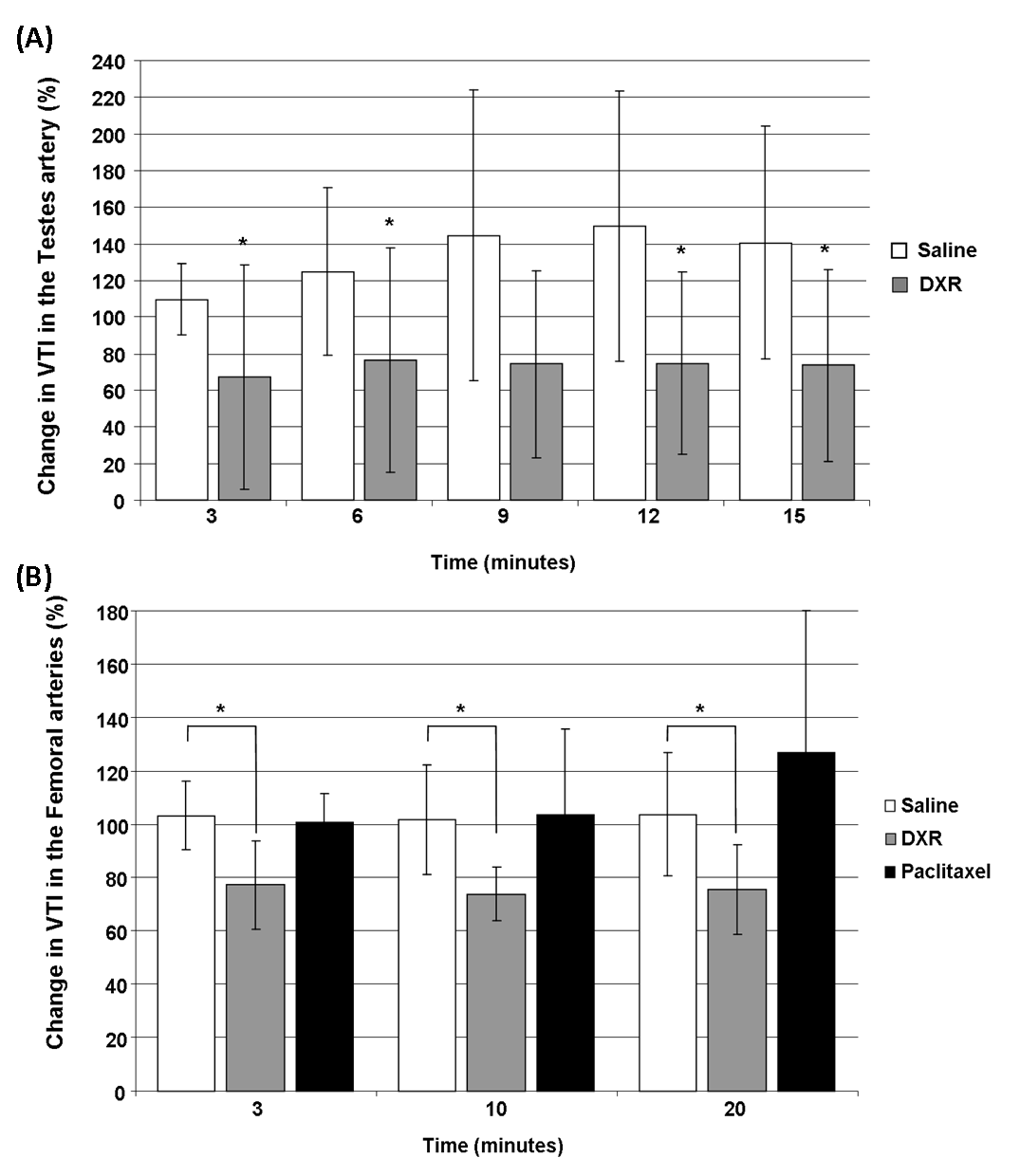

Supplement: Figure S2 — Pulse-wave Doppler measurement of testicular and femoral arterial blood flow. Using PW Doppler mode, blood flow was measured and quantified by analyzing Velocity-Time Integral (VTI) using the appropriate VisualSonics software. PW Doppler blood flow was continuously monitored before and following doxorubicin administration. Results are presented as mean±SD of non-normalized values (saline injected mice were not standardized as 100%). The data obtained from the images as presented in figure 1A, was presented as an Excell curve provided by the VisualSonics software. A. Graphic representation of testicular blood flow 3, 6, 9, 12 and 15 minutes after doxorubicin (n = 8) or saline (n = 7) injection indicating a rapid and constant fall in testicular blood flow of doxorubicin treated mice (* P<0.01). B. Graphic representation of femoral blood flow volume 3, 10 and 20 minutes after doxorubicin (n = 12), Paclitaxel (n = 14) or saline (n = 10) injection indicating a rapid and constant fall in femoral blood flow of doxorubicin treated mice (* P<0.01). (TIF) [file pone.0023492.s002.tif]
